# Visions for Future

**DOI:** 10.5681/joddd.2009.001

**Published:** 2009-03-16

## Editorial

In the third volume of JODDD, we have brought a number of changes to the journal. The layout of the pages has changed to accommodate for information on online access to articles on the first page, and also the new licensing of articles, which are now under a Creative Commons Attribution 3.0 License. This approach is taken to further keep in line with open access publishing. With the type of the articles identified on the first page of each article, we are placing more emphasis on other types of materials that we are planning to include in our journal—letters to the editor, commentaries, short communications—in addition to original articles and case reports that we publish.

We are pleased to announce that starting with our third volume (2009), Journal of Dental Research, Dental Clinics, Dental Prospects is covered by Elsevier’s Expert Medica database for healthcare professionals (EMCare). With higher visibility for the published articles, authors will enjoy a broader audience for their articles. This will definitely make the journal more attractive to authors to publish their work in our journal. In fact, as we move forward and the journal is firmly established, we witness an increasing number of submissions to the journal, and we hope this will eventually lead to more quality material being published. This in fact means more editorial workload and a need for a broader pool of voluntary reviewers to undertake the task of peer review. We are trying our best to prevent delays in the review and editing processes in order to timely publish research material. However, without the help of our editorial board members and external reviewers, such a mission would be impossible. We are asking every interested specialist to register with our journal’s online system at our website as reviewers and let us approach them with a submission to review. We hereby have to thank the members of our editorial board and all the reviewers that undertook the task of reviewing manuscripts during 2008.

New proposals for treatment modalities, patient care, dental materials, equipment, and techniques are definitely welcome in our journal to make up the “prospects” section of the journal. We would like to see such a section become a useful forum for dental practitioners from all over the globe. While other sections will focus on articles of already established subjects, this forum will attempt to stay at the cutting edge, providing a think tank for future research proposals.

In this issue, we have articles from almost all disciplines of dentistry, and although this implies a general attitude for the journal, this trend can attract more specialized readership to certain articles in our journal. We would like to receive comments from specialists on certain articles of interest and we would be pleased to publish those commentaries under a special section to facilitate scholarly communication. This will also help the general dental practitioners’ vision on various aspects of prevention, treatment and rehabilitation. We are inviting all dental practitioners from all over the world, to share with us their comments and points of view, which will definitely help us in shaping a highly prestigious journal.

